# Long-term Follow-up After Hypothermic Oxygenated Machine Perfusion in DCD Liver Transplantation: Results of a Randomized Controlled Multicenter Trial (DHOPE-DCD)

**DOI:** 10.1097/SLA.0000000000006876

**Published:** 2025-08-05

**Authors:** Rianne van Rijn, Chikako Endo, Efrayim H. Küçükerbil, Hans Blokzijl, Joris Blondeel, Miriam Cortes Cerisuelo, Minneke J. Coenraad, Sarwa Darwish Murad, Michail Doukas, Hasan Eker, Robbert J. de Haas, Volkert A.L. Huurman, Vincent E. de Meijer, Diethard Monbaliu, Ivo J. Schurink, Jules J.G. Slangen, Wojciech G. Polak, Jeroen de Jonge, Robert J. Porte

**Affiliations:** * Department of Surgery, Section Hepatobiliary Surgery and Liver Transplantation, University of Groningen, University Medical Center Groningen, Groningen, The Netherlands; † Erasmus MC Transplant Institute, Department of Surgery, Division of HPB and Transplant Surgery, Erasmus University Medical Center, Rotterdam, The Netherlands; ‡ Department of Gastroenterology and Hepatology, University of Groningen, University Medical Center Groningen, Groningen, The Netherlands; § Abdominal Transplantation, KU Leuven, University Hospitals Leuven, Leuven, Belgium; ‖ Institute of Liver Studies, Kings College Hospital NHS Foundation Trust, London, UK; ¶ Department of Gastroenterology and Hepatology, Transplant Center, Leiden University Medical Center, Leiden, The Netherlands; # Department of Gastroenterology and Hepatology, Erasmus Transplant Institute, Erasmus University Medical Center, Rotterdam, The Netherlands; ** Department of Pathology, Erasmus University Medical Center, Rotterdam, The Netherlands; †† Department of Transplant Surgery, Ghent University Hospital, Ghent, Belgium; ‡‡ Department of Radiology, University of Groningen, University Medical Center Groningen, Groningen, The Netherlands; §§ Department of Surgery, Transplant Center, Leiden University Medical Center, Leiden, The Netherlands

**Keywords:** acute rejection, donation after circulatory death, hypothermic machine perfusion, liver transplantation, long-term follow-up, nonanastomotic biliary strictures, static cold storage

## Abstract

**Background and Aim:**

Transplantation of livers from donation after circulatory death (DCD) donors is associated with an increased risk of nonanastomotic biliary strictures (NAS). Dual hypothermic oxygenated machine perfusion (DHOPE) of donor livers before transplantation has been shown to reduce the incidence of symptomatic NAS and acute cellular rejection (ACR) within 6 months, but long-term results are unknown. The aim of this study was to assess the 5-year incidence of NAS and ACR in the DHOPE-DCD Trial (ClinicalTrials.gov number NCT02584283).

**Methods:**

Between January 2016 and July 2019 recipients of DCD livers in 6 European centers were randomly assigned to receive that liver either after DHOPE (machine perfusion group) or after conventional static cold storage (control group). Primary endpoint was the incidence of NAS at 5-year follow-up. Secondary endpoints included ACR, graft, and patient survival.

**Results:**

A total of 78 patients were included in the machine perfusion group and 78 patients in the control group. After 5 years of follow-up, the incidence of NAS was significantly lower in the machine perfusion group, compared with control group: 14% versus 26% (hazard ratio: 0.47, 95% CI: 0.23–0.99; *P*=0.048). In patients with immune-mediated disease, who are at increased risk of ACR, the rate of ACR was significantly lower in the machine perfusion group: 0% versus 32% (*P*=0.036).

**Conclusions:**

The short-term benefits of DHOPE in DCD liver transplantation persist up to 5-year post-transplant, with significant reductions in incidence of NAS, and ACR in high-risk patients, compared with conventional static cold storage.

Donor organ shortage has led to increased use of extended criteria donor liver grafts, such as livers obtained from donation after circulatory death (DCD) donors.^1^ Although DCD has helped to increase the number of donor livers for transplantation, DCD liver transplantation comes at a price. Nonanastomotic biliary strictures (NAS) occur 3 times more often after DCD liver transplantation than after donation after brain death liver transplantation.^2,3^ Ischemia-reperfusion injury (IRI) associated with the additional period of warm ischemia in DCD donors has been identified as the main cause of biliary injury and NAS after DCD liver transplantation.^2,3^

(Dual) hypothermic oxygenated machine perfusion (D)HOPE has been acknowledged as a novel preservation method that reduces the risk of IRI-related complications, especially after transplantation of extended criteria donor liver grafts.^4–9^ The efficacy of hypothermic oxygenated machine perfusion has been demonstrated in several recently published randomized controlled trials.^4–9^ These studies have identified a reduction in the incidence of NAS, post-reperfusion syndrome, and early allograft dysfunction. The DHOPE-DCD Trial was a European multicenter, randomized controlled trial that evaluated the efficacy of DHOPE in recipients of a DCD liver graft. In this trial, a significant reduction of 68% in the risk of symptomatic NAS within 6 months after transplantation was noted in DCD livers that underwent DHOPE, compared with livers that underwent only static cold storage (SCS). Moreover, the short-term outcome data of this trial suggested a 2-fold reduction in the incidence of acute cellular rejection (ACR) after DHOPE, compared with static cold storage.^5^ Experimental studies have indicated that the IRI-mitigating effect of DHOPE results in a less immunogenic environment in the donor liver, which could explain the reduced risk of rejection.^10^

Although the impact of DHOPE on short-term (6–12 months) outcomes after liver transplantation is well documented, it remains unclear whether this effect persists with longer follow-up. Recent observational studies have suggested that a number of new cases of NAS may develop after the first 6 months post-transplantation.^11^ The current study, therefore, aimed to assess the long-term outcome of hypothermic oxygenated perfusion in DCD liver transplantation in a randomized clinical trial. We analyzed the 5-year incidence of NAS and the occurrence of ACR after transplantation of DCD livers who received DHOPE compared with SCS alone.

## MATERIALS AND METHODS

### Trial Design

The DHOPE-DCD Trial (ClinicalTrials.gov number NCT02584283) was an investigator-initiated multicenter randomized controlled trial. Detailed description of the trial design and protocol was previously published.^5,12^ In summary, patients receiving a DCD liver transplantation were included and randomly assigned 1:1, to receive a liver graft either after DHOPE or after SCS alone, using block randomization stratified for trial site and primary sclerosing cholangitis (PSC) as indication for transplantation. In the intervention group, end-ischemic DHOPE was performed in the recipient hospital, after the liver graft was procured and preserved with conventional SCS. Perfusion was performed for at least 2 hours using the Liver Assist (XVIVO, Gothenburg, Sweden) with oxygenated Belzer machine-perfusion solution (Bridge to Life, Northbrook, IL).^5^ Post-transplant, patients received care as usual in the participating centers. Immunosuppressive medication consisted of induction with basiliximab and maintenance of tacrolimus (either with or without mofetil mycophenolate) and a rapid taper of steroids.

The research ethics committees at each trial site approved the protocol of the original and the current study. Patients gave written informed consent for the original trial and this was renewed for the current study. The trial was designed and conducted based on principles of good clinical practice guidelines (International Conference on Harmonization-Good Clinical Practice), Declaration of Helsinki, and Declaration of the Consolidated Standards of Reporting Trials (CONSORT) statement.

Primary endpoint in the present study was the incidence of symptomatic NAS at 5 years after transplantation. NAS was defined as the occurrence of any irregularity or narrowing of the lumen of the intrahepatic or extrahepatic donor bile ducts, excluding the biliary anastomosis, in combination with clinical symptoms (eg, jaundice or cholangitis) or elevation of cholestatic laboratory values, in the presence of a patent hepatic artery. Per protocol, magnetic resonance cholangiography (MRC) imaging was performed at 6 months post-transplantation in all recipients to minimize reporting bias by local trial sites and allow for objective assessment by an Adjudication Committee consisting of independent radiologists blinded for study group assignment. Secondary endpoints included treatment for NAS, graft and patient survival, acute and chronic rejection.

The diagnosis of ACR was based on biopsy confirmation and/or a positive clinical response after (increased) immunosuppressive treatment. A subgroup analysis was performed in patients with immune-mediated liver diseases, including those with PSC, primary biliary cholangitis, and autoimmune hepatitis as primary indication for liver transplantation. The rejection activity index (RAI) score was scored by a specialist pathologist, blinded for study group, and quantified the histopathologic severity of rejection.^13^

### Statistical Analysis

The primary endpoint was analyzed using a Cox proportional hazards regression model with calculation of hazard ratios (and 95% CI), after correction for predefined risk factors, as described in the statistical analysis plan.^5^ The model was corrected for stratification factors used for the block randomization (trial site and PSC) and for prespecified donor-specific risk factors (donor risk index and donor warm-ischemia time, defined as the time between circulatory arrest and in situ cold flush-out). Secondary endpoints, such as incidence of ACR, were analyzed using Cox-regression modeling with stratification factors as covariates or log-rank test. All time-to-event outcomes were presented as Kaplan-Meier curves. Mann-Whitney *U* test was used to compare continuous data and χ^2^ or Fisher exact test for categorical data. All tests were 2-sided, and *P*-values of <0.05 were considered statistically significant. Analyses were performed with SPSS software (version 28.0).

## RESULTS

A total of 245 patients were assessed for eligibility, of which 156 patients were included in the trial, 78 patients in the machine perfusion group and 78 patients in the control group. Baseline characteristics were all well-matched (Supplementary Table 1, Supplemental Digital Content 1, http://links.lww.com/SLA/F577). Follow-up period of 5 years was complete for all patients, except for 3 patients who were lost to follow-up at 1.9, 3.7, and 3.8 years at which point they were alive with a functioning graft. There was no withdrawal of consent.

### Symptomatic Nonanastomotic Biliary Strictures

**FIGURE 1:**
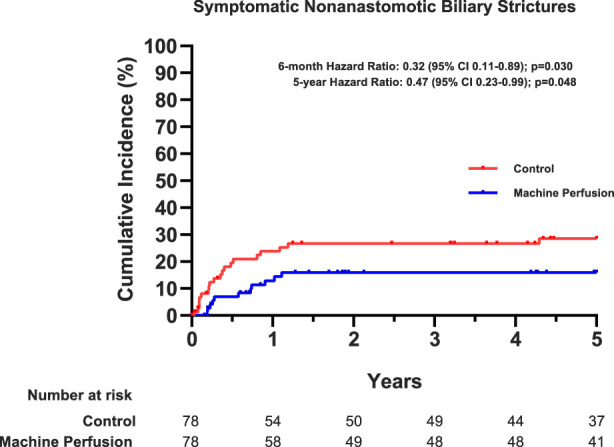
Cumulative incidence of symptomatic nonanastomotic biliary strictures. Shown are the time-to-event Kaplan-Meier curves for symptomatic nonanastomotic biliary strictures up to 5 years after liver transplantation. Events are censored for patient death and graft loss for other reasons than symptomatic nonanastomotic biliary strictures. The hazard ratio was adjusted for stratification factors (transplantation center and primary sclerosing cholangitis) and for prespecified, established donor risk factors (donor warm-ischemia time and donor risk index); the *P*-values are from a Cox-regression analysis.

At 5 years after transplantation, the incidence of symptomatic NAS was significantly lower in the machine perfusion group compared with the control group: 11 of 78 patients (14%) in the machine perfusion group versus 20 of 78 patients (26%) in the control group (hazard ratio: 0.4, 95% CI: 0.23–0.99; *P*=0.048). As can be deducted for the Kaplan-Meier curves depicted in Figure 1, most cases of NAS developed within the first 12 months post-transplantation, after which the incidence curves for both groups run almost parallel.

The clinical symptoms, laboratory abnormalities and interventions for each patient with NAS are specified in Table 1. The cumulative number of treatments for NAS was lower in the machine perfusion group than in the control group (81 vs 120, respectively) (Table 2).

**TABLE 1:** Clinical Symptoms, Laboratory Abnormalities, and Biliary Interventions in Patients With Symptomatic Nonanastomotic Biliary Strictures*

		Cholestatic laboratory tests at time of detection of NAS†			No. NAS-related biliary treatments/interventions			
	Days between transplant and first signs of NAS	Gamma glutamyl transferase (U/L)	Alkaline phosphatase (U/L)	Bilirubin (µmol/L)‡	Antibiotics for cholangitis	Endoscopic or percutaneous stenting	Reoperation for NAS	Death due to NAS
Machine perfusion group								
1	94	149	316	30	1	—	Retransplant for NAS	
2	70	219	222	157	1	3	—	Death
3	83	91	217	30	>10	>10	(Unfit for retransplant)	
4	102	263	378	10	1	6	—	Death
5	71	743	547	9	1	1	—	
6	373	378	401	34	4	8		
7	270	148	251	7	1	>10		
8	210	223	523	284	2	3		Death
9	265	265	438	17	—	—		
10	406	229	238	56	7	3	Retransplant for NAS	
11	331	162	331	92	0	3	Retransplant for NAS	
Total no. events	28	47	3	3				
Control group								
12	34	991	754	40	1	—	—	
13	28	338	962	320	1	—	Retransplant for NAS	
14	12	63	157	8	—	4	Resection EHBD§	
15	81	832	1150	31	3	>10	Retransplant for NAS	
16	176	618	650	30	—	1	—	
17	34	1091	250	48	4	2	Retransplant for NAS	
18	129	695	653	30	4	5	—	Death
19	147	226	733	22	3	3	Retransplant for NAS	
20	137	1430	1065	77	1	9	—	Death
21	43	366	323	19	4	1	—	
22	76	189	144	9	1	4	—	
23	35	370	272	19	3	4	—	
24	100	560	317	200	2	4	Retransplant for NAS	
25	78	2801	4017	376	1	1	Retransplant for NAS	
26	187	988	337	12	—	6		
27	311	80	245	19	2	10	Listing for retransplant	
28	1567	223	256	12	2	—		
29	397	51	83	9	—	—		
30	434	54	98	6	6	—		
31	295	202	988	46	2	7		Death
Total no. events					40	71	8	3

* All patients had radiologically confirmed nonanastomotic strictures of the donor bile ducts and in the presence of a patent hepatic artery. Interventions for other types of biliary complications, such as anastomotic strictures, are excluded from this overview.

† Laboratory results were based on the nearest available predefined sample point.

‡ To convert results for serum bilirubin from µmol/L to mg/dL, divide results by 17.1.

§ Patient required resection of necrotic EHBD with multiple strictures of the intrahepatic bile ducts on cholangiography.

EHBD indicates extrahepatic bile duct.

**TABLE 2:** Primary and Secondary Outcomes^*^

Outcome	Machine perfusion (N=78)	Control (N=78)	*P*
Primary endpoint			
Nonanastomotic biliary strictures, n (%)^†^			
At 6 mo	5 (6)	14 (18)	0.030
At 5 yrs	11 (14)	20 (26)	0.048
Secondary endpoints			
Treatment or interventions for NAS, n^‡^	81	120	
Endoscopic or percutaneous dilatation and stenting	47	71	
Antibiotic treatment for cholangitis	28	40	
Retransplantation	3	6	
Death due to NAS	3	3	
Croome classification for patients with NAS, n (%)^§^			0.327
Minor form	1 (9)	7 (35)	
Confluence dominant	3 (27)	5 (25)	
Diffuse necrosis	5 (45)	3 (15)	
Multifocal progressive	2 (18)	5 (25)	
Biliary anastomotic strictures, n (%)	35 (45)	37 (47)	0.748
Patients with both NAS and anastomotic strictures	9 (12)	15 (19)	0.428
Days between anastomotic stricture and NAS, median (IQR)	209 (88–315)	51 (35–108)	0.053
Biliary leakage within 6 months, n (%)	6 (8)	9 (12)	0.415
Acute cellular rejection, n (%)^‖^	7 (9)	12 (15)	0.221
Acute cellular rejection in immune-mediated disease patients, n (%)	0/12 (0)	7/22 (32)	0.052
Primary sclerosing cholangitis	0/7	6/13	
Primary biliary cholangitis	0/5	1/8	
Autoimmune hepatitis	0/0	0/1	
Days between transplantation and acute rejection, median (IQR)	9 (4–65)	56 (9–239)	0.120
Biopsy proven acute rejection, n	4	11	0.117
Histopathological severity (RAI)^¶^, median (IQR)	6 (4–7)	6 (4–7)	1
Borderline (3)	1	1	
Mild (4–5)	1	3	
Moderate (6–7)	2	7	
Severe (8–9)	0	0	
Treatment for acute rejection, n			0.980
Tacrolimus only	1	1	
Steroid only	4	7	
Tacrolimus and steroid	1	2	
Steroid + anti-thymocyte globulin	1	2	
Chronic rejection, n (%)	0 (0)	2 (3)	0.497
Complications Clavien-Dindo grade ≥3 within 1 yr—no. patients, n (%)	39 (50)	41 (53)	0.749
Retransplantation within 5 yrs, n (%)	10 (13)	12 (15)	0.753
Nonanastomotic biliary strictures	3 (4)	6 (8)	
Primary nonfunction^#^	0	1 (1)	
Hepatic artery thrombosis	2 (3)	1 (1)	
Severe liver laceration^**^	0	2 (3)	
Secondary liver dysfunction in the setting of multiorgan failure of unknown origin	1 (1)	0	
Rejection	1 (1)	1 (1)	
Recurrent liver disease	3 (4)	1 (1)	
Patient death within 5 yrs, n (%)	29 (37)	23 (29)	0.350
Malignancy	7 (9)	10 (13)	
Infection/sepsis	5 (6)	6 (8)	
Cardiovascular	6 (8)	3 (4)	
Multiorgan failure	2 (3)	0	
Non-anastomotic biliary strictures	3 (4)	3 (4)	
Hemophagocytic syndrome	1 (1)	0	
Pulmonary fibrosis	1 (1)	0	
Other^††^	4 (5)	1 (1)	

^*^ Continuous data are presented as median and interquartile range (IQR), unless indicated otherwise.

^†^ The *P*-value is from Cox-regression analyses adjusted for prespecified covariates, including stratification factors (transplantation center and primary sclerosing cholangitis) and established donor risk factors (donor warm-ischemia time and donor risk index).

^‡^ Cumulative number of interventions for nonanastomotic biliary strictures and treatments of related complications, such as cholangitis. Number of events was truncated at 10 if patients underwent more than 10 interventions.

^§^ Croome classification was used to classify the radiological patterns of NAS.^14^

^‖^ Excluding 2 patients in the machine perfusion group and 5 in the control group who had subtherapeutic or no immunosuppressive treatment because of an infection or chemotherapy treatment. Continuous data are presented as median and interquartile range (IQR).

^¶^ RAI denotes rejection activity index, based on the diagnostic triad of portal inflammation, bile duct damage, and venous endothelial inflammation, graded from 0 to 9 points, which was used to define severity of acute cellular rejection.

^#^ Primary nonfunction was defined as liver failure requiring retransplantation or leading to death within 7 days after transplantation without any identifiable cause such as hepatic artery thrombosis, portal vein thrombosis, or acute rejection.

^**^ Liver laceration occurred during donor hepatectomy and caused severe bleeding and subcapsular hematoma after reperfusion in the recipient, necessitating gauze packing and listing for retransplantation.

^††^ Other cause of death was massive hemorrhage during retransplantation, non-compliance to immunosuppressive medication, alcoholic liver cirrhosis in donor liver graft, euthanasia due to arthritis in machine perfusion group, and recurrent metabolic dysfunction-associated fatty liver disease (MAFLD) cirrhosis in control group.

### Clinical Evolution of Abnormalities on Protocol MRC

In total, 133 of 156 patients (85%) had an MRC at 6 months after transplantation of which 19 patients had biliary abnormalities on MRC in combination with clinical symptoms of NAS. Of the remaining 114 asymptomatic patients, 40 patients had no abnormalities and 74 patients had biliary abnormalities on MRC. Of the 74 patients with asymptomatic biliary abnormalities on MRC, 10 patients (14%) developed symptomatic NAS, 5 out of 43 (12%) in the machine perfusion group and 5 out of 31 (16%) in the control group. Of the 40 patients without abnormalities on MRC and without symptoms, 2 out of 40 (5%) developed symptomatic NAS, one in each study group (Supplementary Figure 1, Supplemental Digital Content 1, http://links.lww.com/SLA/F577).

### Acute Cellular Rejection

**FIGURE 2:**
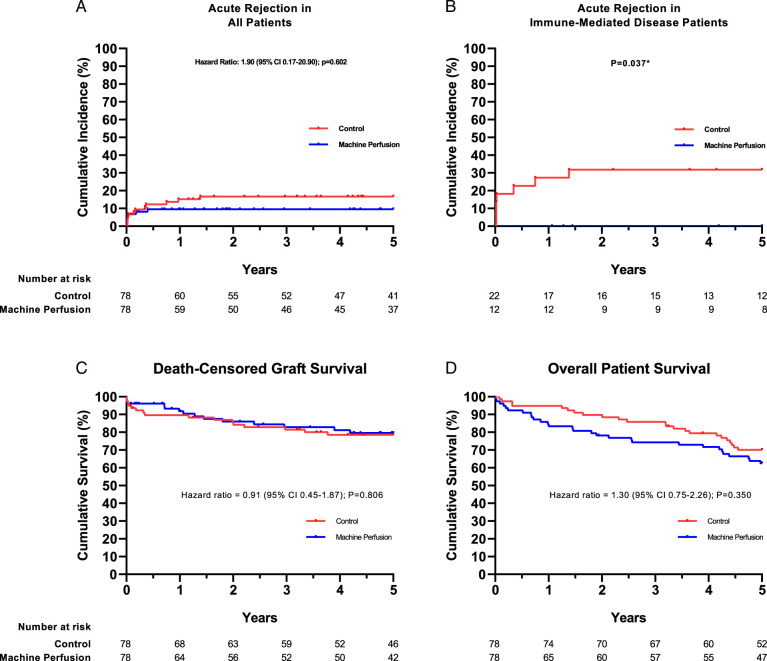
Cumulative incidence of acute cellular graft rejection, death-censored graft survival and overall patient survival. Shown are the time-to-event Kaplan-Meier curves for acute cellular graft rejection up to 5 years after liver transplantation. Events are censored for patient death and graft loss for other reasons than acute graft rejection. Data are shown for all patients (A) and for the subgroup of patients (B) who underwent liver transplantation for an immune-mediated liver disease (primary sclerosing cholangitis, primary biliary cholangitis, and autoimmune hepatitis). Shown are the time-to-event Kaplan-Meier curves for death-censored graft survival (C) and overall patient survival (D) up to 5 years after liver transplantation. Shown are hazard ratio’s and *P*-values from a Cox-regression analysis, except for (B), where the *P*-value is based on a log-rank test as Cox-regression analysis was not possible due to 0 events in the machine perfusion group.

Up to 5 years post-transplant, 19 patients experienced ACR: 7 (9%) in the machine perfusion group versus 12 (15%) in the control group (*P*=0.221; Table 2). In patients who underwent transplantation for an immune-mediated liver disease, who have a higher a priory risk of rejection, the incidence of ACR was significantly lower in the machine perfusion group [0 out of 12 (0%)], compared with controls [7 out of 22 (32%), *P*=0.036] (Table 2). The incidence of ACR in relation to time after transplantation is depicted in Figure 2A-B.

The histopathologic severity of rejection, as reflected by the median RAI score, was comparable in both groups (Supplementary Figure 2). The most common treatment consisted of boluses of steroids: 57% in the machine perfusion group and 58% in control group. In both groups, there was one case where severe and refractory ACR led to retransplantation (Table 2).

### Patient and Death-censored Graft Survival

The 5-year death-censored graft survival was similar in both groups, 82% versus 79% (*P*=0.806, Fig. 2C). Retransplantation due to NAS occurred 3 times in the machine perfusion group versus 6 times in the control group (Table 2). Patient survival at 5 years was comparable between the groups (63% machine perfusion vs 70% control group, *P*=0.254, Fig. 2D). Malignancy was the leading cause of death, followed by infectious causes and/or sepsis (Table 2).

## DISCUSSION

The current study aimed to determine the long-term outcomes of the DHOPE-DCD Trial in which patients undergoing DCD liver transplantation were randomly assigned to receive that liver after preservation with either DHOPE or conventional SCS (controls). At 5 years of follow-up, the risk of symptomatic NAS was 2 times lower in the machine perfusion group, compared with the control group: 14% versus. 26% respectively (*P*=0.048). In patients with immune-mediated disease, who are at increased risk of ACR, the rate of ACR was significantly lower in the machine perfusion group: 0% versus 32% (*P*=0.036). No significant differences were found in 5-year graft or patient survival between the 2 groups.

The short-term postoperative outcomes of the DHOPE-DCD Trial were reported previously and included a lower incidence of early allograft dysfunction (26% vs 40%), lower incidence of post-reperfusion syndrome (12% vs 27%), and lower incidence of symptomatic NAS (6% vs 18%) at 6 months post-transplantation in the machine perfusion group compared with controls.^5^ It remained to be determined whether this positive effect persisted with longer follow-up. In the present study, patient follow-up was extended up to 5 years in which a total of 12 new cases of NAS were observed (6 in each group) with incidence curves practically running parallel after 12 months. Overall, the impact of DHOPE on the development of NAS remained clinically and statistically significant up to 5 years post-transplant. This observation indicates that the initial observation period of 6 months was valid and representative for the protective effect of DHOPE on the development of NAS.

An unexpected finding at 6 months of follow-up was the relatively high number of patients with asymptomatic biliary abnormalities on protocol MRC.^5^ With follow-up up to 5 years, it became evident that 14% of all patients with asymptomatic biliary abnormalities on protocol MRC at 6 months, later developed symptoms of jaundice, cholestasis or cholangitis, yet 86% remained asymptomatic. The incidence of new cases of symptomatic NAS between 6 months and 5 years was 12% in the machine perfusion group and 16% in the control group. Almost all new cases presented within 1 year after transplantation and the incidence curves ran almost horizontal thereafter. Interestingly, also in patients who did not have any biliary abnormalities on the 6-month protocol MRC 5% later developed symptomatic NAS. The protective effect of DHOPE on the development of NAS seemed to be most pronounced in the initial 6 months after transplantation. This finding suggests that biliary imaging with MRC at 6 months after transplantation has been a reliable method to assess the role of DHOPE in the prevention of NAS after DCD liver transplantation.

In a ‘real-world’ large observational, multicenter study on hypothermic machine perfusion (HOPE-REAL study), Eden et al^11^ recently reported an incidence of NAS after DCD liver transplantation of 15% at 2 years and 20% at 5 years after transplantation. The results of the current study are slightly favorable compared with those of the HOPE-REAL study. Although these data reflect a consistent and favorable 2-fold reduction in the risk of NAS in DCD liver transplantation after hypothermic machine perfusion, compared with SCS alone, it also indicated that NAS still occurs in ~1 out of 10 patients. Lower NAS rates (as low as 3%) have been reported after other machine perfusion methods, such as normothermic machine perfusion (NMP) after DHOPE or normothermic regional perfusion in the DCD donor before organ procurement.^15–17^ However, a direct comparison between these preservation modalities is hampered by the fact that both NMP and NRP result in a non-utilization rate of about 30%, as livers that fail the viability testing during NMP or NRP are declined for transplantation. In the present study, no viability testing was performed during machine perfusion, and no grafts were discarded during or after perfusion. Although testing of DCD livers (including standard, low-risk DCD livers) during either NMP or NRP could further lower the risk of NAS, it will also reduce the utilization rate and thus reduce the pool of transplantable donor livers, especially as the vast majority of symptomatic NAS patients remained with a functioning graft. Apart from this, NMP is technically more challenging and more expensive than hypothermic machine perfusion. In a recent economic analysis, it was demonstrated that DHOPE is cost-effective, when considering all medical costs up to 1 year after transplantation.^18^ In the simplest cost scenario, considering only costs for machine perfusion equipment and disposables, DHOPE was cost-effective after just one procedure.^17^ Recent clinical evidence also indicates that DHOPE can be used to safely prolong the preservation time, helping to improve transplantation logistics.^19,20^ The efficacy of hypothermic machine perfusion can be enhanced further by inclusion of viability tests, such as the measurement of the mitochondrial injury marker flavin mononucleotide measurement (FMN) in the perfusion fluid.^20^ Although the diagnostic value of FMN was recently validated in a large international multicenter study, the best cutoff value still remains to be determined.^13,21^

The observed impact of DHOPE on the risk of ACR after transplantation is interesting and supports previous experimental studies suggesting that the reduction of IRI with hypothermic oxygenated machine perfusion may contribute to a less immunogenic environment, making donor livers less prone to rejection.^22,23^ The incidence of ACR in the machine perfusion group (10%) was similar to that reported earlier by Eden et al^13^ and was lower than in the control group (17%), although this did not reach statistical significance. However, in patients with an immune-mediated liver disease, the incidence of ACR was significantly lower in the machine perfusion group, compared with the control group (0 vs 32%; *P*=0.036). This is an interesting finding as this group of patients is known to have an increased risk of developing ACR, with reported incidences up to 57% within the first year after transplantation.^24^ The current clinical findings support previous studies suggesting that hypothermic machine perfusion may have an immunomodulatory effect on the graft and thereby lower the risk of ACR.^10^ Nevertheless, it should be noted that this observation is based on a subgroup with a relatively small number of patients. Therefore, this data needs to be confirmed in other studies and more research in the exact underlying mechanisms will be necessary.

In summary, this is the first randomized controlled trial that provides data on the long-term effect of DHOPE in DCD liver transplantation. This technique not only improves short-term postoperative outcomes, with lower incidence of post-reperfusion syndrome and early allograft dysfunction, but also significantly improves long-term results at 5 years after transplantation with a 2-fold lower incidence of NAS and excellent graft survival rates. In addition, this study suggests that recipients who are at increased risk of ACR, such as patients with immune-mediated diseases, may have an additional benefit of DHOPE, as it significantly lowers their risk of ACR. These findings underscore the long-term benefits of DHOPE and add to the evidence that DHOPE is a highly effective preservation method for DCD liver grafts.

## Supplementary Material

## DISCUSSANTS

### Constantino Fondevila Campo (Madrid, Spain)

Thank you very much for the kind invitation to discuss this paper, and congratulations on an excellent presentation and to all the hospitals involved. I have organized my questions according to the main clinical endpoints discussed, even though, as a side comment, we should all include patient-reported outcomes in future clinical trials, as we learned in the Special Lecture presented by Professor Clavien’s group.

I have the following comments and questions:

First, Biliary Strictures: This remains the Achilles heel of liver transplantation, particularly in the context of DCD donor grafts. I found it striking, upon reviewing this paper and revisiting the landmark study published in the *New England Journal of Medicine* in 2021, how high the rate of biliary strictures you showed was.

Why are 5-year rates of biliary strictures (both nonanastomotic and anastomotic) in the trial higher than those reported in other studies with similar preservation methods, and above controlled donation after circulatory death (cDCD) benchmark values, despite a significant reduction compared with the control group?

Could you please clarify what the surveillance and follow-up protocols were between 6 months and 5 years post-transplant, particularly whether protocol MRCPs were routinely performed? Could you also explain how clinically silent biliary complications could have been detected without standardized imaging?

Do you see potential to identify predictive factors–either pre-procurement or during the DHOPE procedure–that differentiate early versus late nonanastomotic biliary strictures, and could this justify switching to normothermic or controlled oxygenated rewarming perfusion techniques in certain cases?

Second, acute cellular rejection (ACR) in primary sclerosing cholangitis (PSC) recipients. You describe less ACR among DHOPE livers transplanted in recipients with immune-mediated disease processes; this observation is primarily based on more ACR among PSC recipients in the control group. What could explain the relatively high rate of acute cellular rejection (ACR) only among PSC recipients in the control group, and were there any center effects or differences in post-transplant immunosuppression management that might have influenced this outcome?

Finally, Patient Survival: How do you explain the low 5-year patient survival rate of 63% in the DHOPE group, and do you believe DHOPE remains a justifiable strategy given this outcome?

### Response From Robert J. Porte (Rotterdam, The Netherlands)

Thank you for your very relevant comments. Coming back to the overall incidence of nonanastomotic biliary strictures, it is indeed a little higher in the control group than what has been reported in benchmark studies. However, the benchmark studies might underestimate the actual incidence of biliary complications, as these were based on retrospective studies, which are known to be subject to bias and patient drop-out. In contrast, the present study is a prospective randomized control trial with a very close follow-up of the patients. As biliary complications were the primary endpoint of the trial, investigators were obviously focused on that. Moreover, the incidence found in the present study was similar to incidences reported in recent observational studies, such as, for example, the HOPE-REAL study that was also mentioned today (incidence of 14%–15% at 2 years; we found 14% at 5 years; the HOPE-REAL even found 20% at 5 years; and 38% anastomotic) (J Hepatol 2025; 82: 97-106).

Regarding testing, in this trial, we didn’t use any biomarkers to assess graft viability or injury during DHOPE, as these were not available at the time. Although the use of biomarkers, such as flavin mononucleotide measurement (FMN), was recently validated in a large international multicenter study, the best cutoff value remains to be determined (J Hepatol 2025; 82: 523-534). In the Netherlands, regular DCD grafts, especially from donors under the age of 60, are not routinely tested for viability using normothermic machine perfusion; they are transplanted immediately after DHOPE. However, if we have older DCD donors of above 60, we do assess them during normothermic machine perfusion. In these cases, we observe a 30% discard rate due to livers failing to meet the biliary viability criteria (Am J Transplant 2022; 22: 1658-1670).

Regarding acute cellular rejection, the numbers are small. It is very difficult to draw definitive conclusions from this, and these kinds of observations need to be confirmed in larger, more dedicated trials. Nonetheless, the high rate of acute cellular rejection seen in the patients with immune-mediated diseases in the control group is in line with rates reported in previous studies (32% and 57%, respectively) (World J Gastroenterol 2012; 18: 1-15).

Finally, regarding 5-year patient survival, 63% is a little lower than benchmark. In the paper, we included a table with the causes of death, and the leading cause was cancer recurrence. For some reason, a relatively high number of HCC patients were included in the trial. That might be an explanation.
