# Supplementary material for: Long-term Follow-up After Hypothermic Oxygenated Machine Perfusion in DCD Liver Transplantation: Results of a Randomized Controlled Multicenter Trial (DHOPE-DCD)
Source: Ann Surg. 2025 Aug 5;282(5):717–24. doi: 10.1097/SLA.0000000000006876 (PMC12513034; doi:10.1097/SLA.0000000000006876)
Supplement: Supplementary file 1 [file sla-282-717-s001.docx]

| Supplementary Table 1. **Characteristics of the Donors and Recipients at Baseline.*** | | | |
| --- | --- | --- | --- |
| **Variable** | **Machine Perfusion**  **(N=78)** | **Control**  **(N=78)** | **P Value** |
| **Donor characteristics** |  |  |  |
| Age – yr | 52 (43-57) | 49 (37-59) | 0.64 |
| Male sex – no. (%) | 52 (67) | 51 (65%) | 0.87 |
| Donor risk index^‡^ | 2.12 (1.84-2.38) | 2.12 (1.86-2.42) | 0.73 |
| Body mass index – kg/m^2^ | 25 (23-27) | 25 (21-28) | 0.95 |
|  |  |  |  |
| **Preservation characteristics** |  |  |  |
| Time from withdrawal of life support to aortic flush-out – min | 29 (22-33) | 27 (21-35) | 0.89 |
| Time from donor circulatory arrest to aortic flush-out – min | 11 (8-13) | 11 (8-15) | 0.38 |
| Static cold ischemia time^§^ – h:min | 6:11 (5:16-6:55) | 6:49 (5:56-7:57) | <0.001 |
| Machine perfusion time – h:min | 2:12 (2:00-2:33) | NA |  |
| Total preservation time^¶^ – h:min | 8:44 (7:46-9:16) | 6:49 (5:56-7:57) | <0.001 |
|  |  |  |  |
| **Recipient characteristics** |  |  |  |
| Age – yr | 60 (52-65) | 60 (52-65) | 0.70 |
| Male sex – no. (%) | 55 (71) | 52 (67) | 0.60 |
| Laboratory MELD score | 14 ( 10-19) | 16 (10-22) | 0.19 |
| Renal replacement therapy | 3 (4) | 2 (3) | 0.65 |

***** Continuous data are presented as median and interquartile range (IQR). NA denotes not applicable, MELD, model of end-stage liver disease.

‡ Donor risk index is a scoring system developed to quantitatively predict the risk of post-transplant graft failure in liver transplantation, based on donor risk factors.^28^

§ Defined as time between aortic cold flush-out in donor to reperfusion in recipient minus machine perfusion time.

¶ Defined as time between aortic cold flush-out in donor to reperfusion in recipient.

| ~~Supplementary Table 2.~~ **~~Diagnosis, Severity and Treatment of Acute Cellular Rejection*~~** | | | |
| --- | --- | --- | --- |
| **~~Variable~~** | **~~Machine Perfusion~~**  **~~(N=78)~~** | **~~Control~~**  **~~(N=78)~~** | **~~P Value~~** |
| ~~Total acute cellular rejection – no.~~ | ~~7~~ | ~~12~~ | ~~0.329~~ |
| ~~Immune-mediated disease patients – no~~ | ~~12~~ | ~~22~~ | ~~0.052~~ |
| ~~Acute cellular rejection – no. (%)~~ | ~~0 (0)~~ | ~~7 (32)~~ | ~~0.036~~ |
| ~~Days between transplantation and rejection – median (IQR)~~ | ~~9 (6-37)~~ | ~~56 (10-169)~~ | ~~0.350~~ |
| ~~Biopsy proven rejection – no.~~ | ~~4~~ | ~~11~~ | ~~0.117~~ |
| ~~Histopathological severity (RAI)~~**^~~‡~~^** ~~– median (IQR)~~ | ~~6 (4-7)~~ | ~~6 (4-7)~~ | ~~1~~ |
| ~~Borderline (3)~~ | ~~1~~ | ~~1~~ |  |
| ~~Mild (4-5)~~ | ~~1~~ | ~~3~~ |  |
| ~~Moderate (6-7)~~ | ~~2~~ | ~~7~~ |  |
| ~~Severe (8-9)~~ | ~~0~~ | ~~0~~ |  |
| ~~Treatment – no.~~ |  |  | ~~0.980~~ |
| ~~Tacrolimus only~~ | ~~1~~ | ~~1~~ |  |
| ~~Steroid only~~ | ~~4~~ | ~~7~~ |  |
| ~~Tacrolimus and steroid~~ | ~~1~~ | ~~2~~ |  |
| ~~Steroid + anti-thymocyte globulin~~ | ~~1~~ | ~~2~~ |  |

~~* Excluding two patients in the machine perfusion group and five in the control group who had subtherapeutic or no immunosuppressive treatment because of an infection or chemotherapy treatment. Continuous data are presented as median and interquartile range (IQR).
‡ RAI denotes rejection activity index, based on the diagnostic triad of portal inflammation, bile duct damage, and venous endothelial inflammation, graded from 0 to 9 points which was used to define severity of acute cellular rejection.~~

**
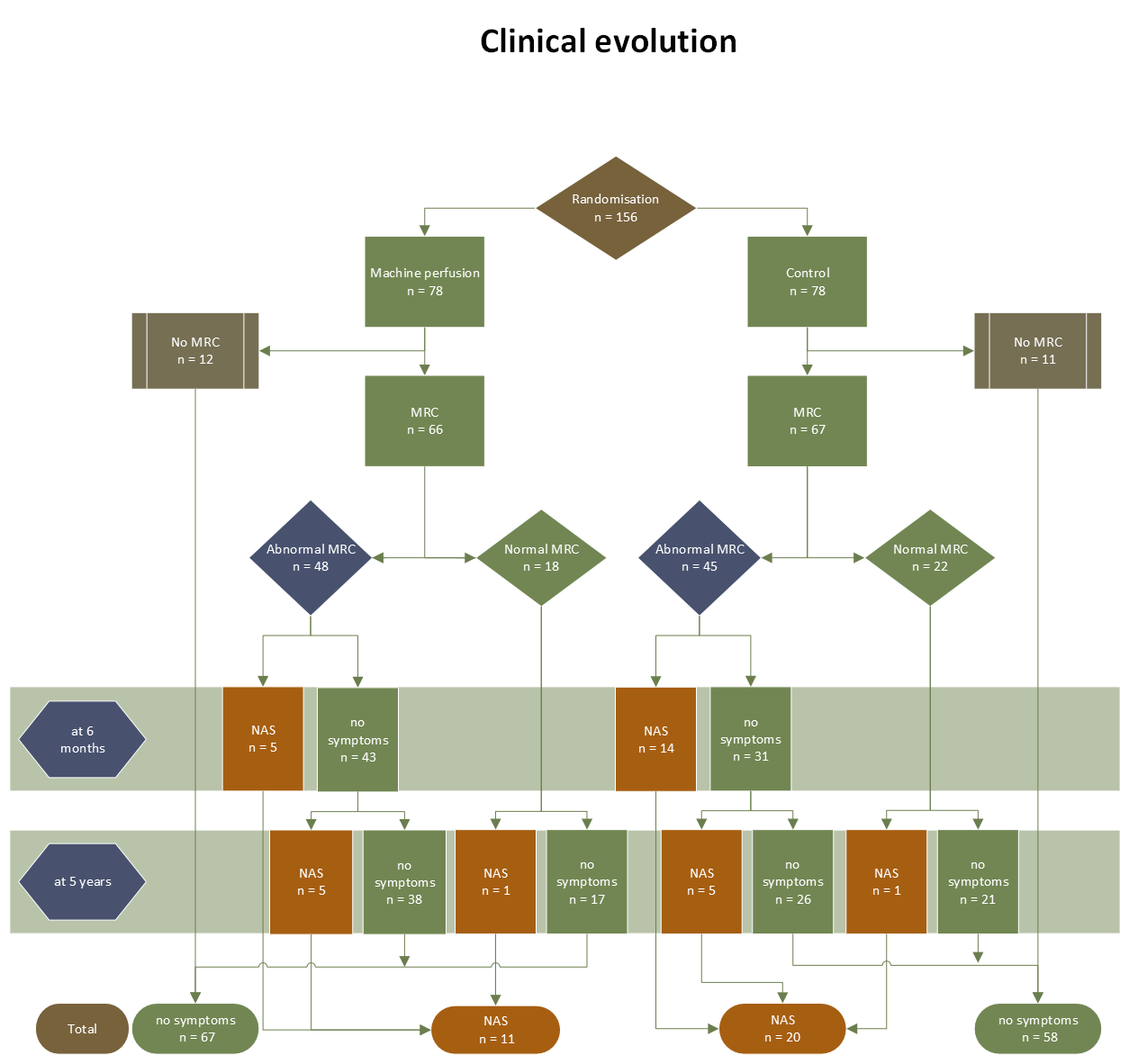
**

**Supplementary Figure 1. Flowchart of Clinical Evolution of Patients with Biliary Abnormalities on Protocol Magnetic Resonance Cholangiography (MRC).**

NAS denotes nonanastomotic biliary strictures.

**Supplementary Figure 2. Rejection Activity Index Score Based on Liver Biopsies in the Machine Perfusion and Control Group.**

Horizontal bars and error bars indicated median and interquartile range.‡ RAI denotes rejection activity index, based on the diagnostic triad of portal inflammation, bile duct damage, and venous endothelial inflammation, graded from 0 to 9 points which was used to define severity of acute cellular rejection.
